# Genome-Wide Identification of Genes Essential for the Survival of *Streptococcus pneumoniae* in Human Saliva

**DOI:** 10.1371/journal.pone.0089541

**Published:** 2014-02-25

**Authors:** Lilly M. Verhagen, Marien I. de Jonge, Peter Burghout, Kiki Schraa, Lorenza Spagnuolo, Svenja Mennens, Marc J. Eleveld, Christa E. van der Gaast-de Jongh, Aldert Zomer, Peter W. M. Hermans, Hester J. Bootsma

**Affiliations:** 1 Laboratory of Pediatric Infectious Diseases, Department of Pediatrics, Radboud University Medical Centre, Nijmegen, the Netherlands; 2 Centre for Molecular and Biomolecular Informatics, Nijmegen Centre for Molecular Life Sciences, Radboud University Medical Centre, Nijmegen, the Netherlands; University Hospital of the Albert-Ludwigs-University Freiburg, Germany

## Abstract

Since *Streptococcus pneumoniae* transmits through droplet spread, this respiratory tract pathogen may be able to survive in saliva. Here, we show that saliva supports survival of clinically relevant *S. pneumoniae* strains for more than 24 h in a capsule-independent manner. Moreover, saliva induced growth of *S. pneumoniae* in growth-permissive conditions, suggesting that *S. pneumoniae* is well adapted for uptake of nutrients from this bodily fluid. By using Tn-seq, a method for genome-wide negative selection screening, we identified 147 genes potentially required for growth and survival of *S. pneumoniae* in saliva, among which genes predicted to be involved in cell envelope biosynthesis, cell transport, amino acid metabolism, and stress response predominated. The Tn-seq findings were validated by testing a panel of directed gene deletion mutants for their ability to survive in saliva under two testing conditions: at room temperature without CO_2_, representing transmission, and at 37°C with CO_2_, representing in-host carriage. These validation experiments confirmed that the *plsX* gene and the *amiACDEF* and *aroDEBC* operons, involved in respectively fatty acid metabolism, oligopeptide transport, and biosynthesis of aromatic amino acids play an important role in the growth and survival of *S. pneumoniae* in saliva at 37°C. In conclusion, this study shows that *S. pneumoniae* is well-adapted for growth and survival in human saliva and provides a genome-wide list of genes potentially involved in adaptation. This notion supports earlier evidence that *S. pneumoniae* can use human saliva as a vector for transmission.

## Introduction

*Streptococcus pneumoniae* is the most common bacterial etiology of community-acquired pneumonia in all ages, and can cause outbreaks in closed settings. The most common manifestations of pneumococcal disease include sinusitis, otitis media, pneumonia and sepsis. The increasing antibiotic resistance and limited serotype coverage of currently available vaccines demonstrates the need for novel approaches in exploring new antimicrobials and vaccines.

All pneumococcal disease begins with the establishment of nasopharyngeal colonization. Once acquired, an individual pneumococcal strain can be carried for weeks to months before its eventual clearance [1]. Pneumococcal carriage induces the production of both mucosal and systemic immunoglobulins. Immunoglobulin G (IgG) and secretory IgA antibodies directed against capsular polysaccharides and surface-associated proteins have been observed in saliva of children in response to colonization with *S. pneumoniae* [2], [3]. The presence of anti-pneumococcal IgA antibodies in saliva has been associated with a lowered risk of pneumococcal acute otitis media (AOM), while the serum IgG antibody level does not seem to be associated with AOM risk [4]. This suggests that the presence of *S. pneumoniae* in saliva is associated with the development of local pneumococcal disease.

Transmission of *S. pneumoniae*, i.e. person-to-person spread, is thought to occur through direct contact with the secretions of colonized individuals. Commonplace activities such as talking, coughing, and sneezing disseminate large amounts of respiratory droplets that subsequently deposit on dry surfaces [5]. These droplets carry bacteria and thus constitute a medium for transmission of *S. pneumoniae*. Understanding of bacterial survival in droplets is crucial for controlling infection transmission via airborne aerosols and/or large droplet routes. Walsh et al. demonstrated that *S. pneumoniae* can survive at ambient temperature and humidity for at least four weeks [6]. For this and other studies investigating the importance of fomites and dry surfaces in microbial transmission (summarized in a systematic review by Kramer et al. [7]), bacteria were suspended in Todd-Hewitt broth supplemented with yeast extract (THY), distilled water or saline solution. To our knowledge, the survival of *S. pneumoniae* in human respiratory secretions has not yet been tested in a laboratory setting. Indirect evidence from studies in humans indicates that *S. pneumoniae* can survive in saliva and that droplets of saliva may be an important source of transmission of the bacterium. *S. pneumoniae* was isolated from saliva of patients with stable chronic obstructive pulmonary disease or asthma [8] and recently also from Dutch children between 5 and 10 years of age [9]. Furthermore, in Israeli army recruits frequent sharing of a drinking glass or bottle was an independent risk factor for pneumococcal carriage, suggesting that transmission of pneumococci may occur via saliva [10].

The aim of the current study was to examine the ability of *S. pneumoniae* to survive and grow in human saliva and to identify the genes essential for its survival in and transmission through saliva *in vitro*. To this end, we examined the behavior of several encapsulated and unencapsulated *S. pneumoniae* strains in human saliva under two experimental conditions: at room temperature (RT) without CO_2_, representing transmission, and at 37°C with CO_2_, representing in-host carriage. Subsequently, *S. pneumoniae* genes essential for survival in saliva under these two conditions were identified using the genome-wide negative selection screenings technology Tn-seq [11]. Finally, the roles of individual genes identified by Tn-seq were validated in single and competitive *in vitro* growth in human saliva.

## Results and Discussion

### Saliva concentration influences *S. pneumoniae* growth and survival

**Figure 1:**
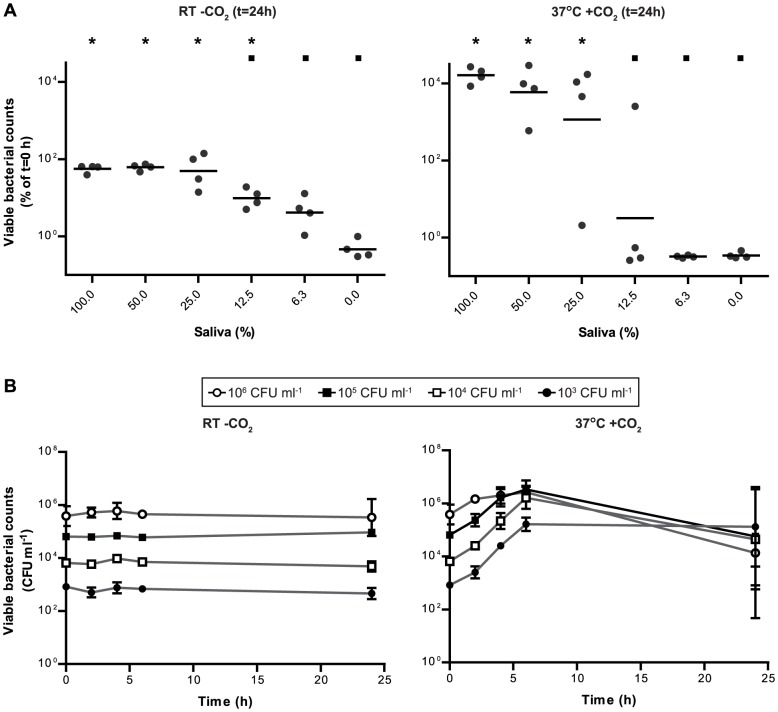
Optimal conditions for growth and survival of *S. pneumoniae* in saliva. A. Growth and survival of *S. pneumoniae* in saliva varies with varying concentrations of saliva. Different dilutions of human saliva in PBS were inoculated with 10^4^ CFU ml^−1^ *S. pneumoniae* Spain^9V^-3 at RT without CO_2_ (left) or at 37°C with CO_2_ (right). After 24 hours of incubation, viable bacterial counts of *S. pneumoniae* were determined and presented as the percentage of the viable bacterial counts detected at t = 0 h. Each point represents a separate replicate. Horizontal lines indicate geometric means. Saliva concentrations for which CFU ml^−1^ significantly differed from 0% saliva by ANOVA and Tukey post-hoc tests are marked with an asterisk (*). Percentages of saliva for which CFU ml^−1^ significantly differed from 100% saliva by ANOVA and Tukey post-hoc tests are marked with a black box (▪). B. Effect of bacterial inoculum size on survival and growth of *S. pneumoniae* Spain^9V^-3 in saliva. Different number of starting CFU were incubated in 100% saliva at RT without CO_2_ (left) or at 37°C with CO_2_ (right) for indicated periods. Data points represent the geometric mean of three replicate experiments. Vertical lines represent the standard deviations of log10 transformed values.

In order to test if *S. pneumoniae* can survive in human saliva, 10^4^ colony forming units (CFU) ml^−1^ of strain Spain^9V^-3 (SP195) were incubated with 100%, 50%, 25%, 12.5%, 6.25% and 0% saliva in phosphate-buffered saline (PBS). Viable bacterial counts at t = 0, t = 4 and t = 24 h post-inoculation were determined for two testing conditions: RT without CO_2_ and 37°C with 5% CO_2_. At RT without CO_2_, *S. pneumoniae* survived in 100% saliva and no significant differences in viable bacterial counts between different saliva concentrations were observed at t = 4 h (not shown). At t = 24 h, the concentration of saliva did significantly affect the survival of *S. pneumoniae* (p<0.01, Figure 1A left). The number of viable counts was significantly lower in 0% saliva (100% PBS) compared with all other concentrations of saliva (all p-values <0.01). Furthermore, viable bacterial counts in 6.25% and 12.5% saliva were significantly lower than bacterial counts in 25%, 50% or 100% saliva, with p-values varying from <0.01 to 0.040. Viable bacterial counts in 25% saliva did not differ significantly from those in 50% or 100% saliva (Figure 1A left panel).

At 37°C with 5% CO_2_, significant differences in viable bacterial counts between different saliva concentrations were observed at t = 4 h (not shown) and at t = 24 h (Figure 1A right panel). The number of bacteria was significantly lower in 0% 6.25% and 12.5% saliva compared with 25%, 50% and 100% saliva at both time points with p-values varying from <0.01 to 0.049. Interestingly, at 37°C *S. pneumoniae* showed growth rather than survival in 100%, 50% and 25% saliva with an increase in the number of bacteria at t = 24 h compared with t = 0 h (Figure 1A, right). This suggests that the pneumococcus is able to feed on and grow in human saliva. Growth will lead to large *S. pneumoniae* cell densities, a condition that may favor transmission of pneumococci through droplet spread.

Because *S. pneumoniae* survival and growth were optimal in undiluted saliva, all subsequently described experiments were performed with 100% human saliva.

### Bacterial inoculum size has no effect on growth and survival of *S. pneumoniae* in saliva

To examine the influence of inoculum size on survival and/or growth of pneumococci in human saliva, growth characteristics of *S. pneumoniae* Spain^9V^-3 were monitored after inoculation of saliva with four different starting inoculae (10^3^, 10^4^, 10^5^ and 10^6^ CFU ml^−1^). Interestingly, the bacterial inocolum size did not lead to major differences in the growth characteristics of *S. pneumoniae* in saliva at either growth condition (Figure 1B). At RT without CO_2_, CFU counts remained near to constant from t = 0 to t = 24 h for all bacterial starting inoculae. For all bacterial starting inoculae growth at 37°C with 5% CO_2_ was observed in the first 6 h after inoculation. In all cases except for 10^3^ CFU ml^−1^, there was a subsequent decline in CFU ml^−1^ between t = 6 and t = 24 h. These results show that at 37°C saliva enables growth of *S. pneumoniae* until reaching a maximum number of viable bacteria, after which growth of *S. pneumoniae* diminishes (Figure 1B right). The maximum number of CFU ml^−1^ was found to be around 10^6^ and was reached faster at higher starting inoculae. A possible explanation for this observation may be that nutrients present in saliva may become scarce as the bacterial population expands until a threshold is reached when not enough nutrients are present for all bacteria to survive.

### Growth characteristics of *S. pneumoniae* in human saliva are strain-specific

The ability to survive and/or grow in saliva was investigated for a selection of *S. pneumoniae* strains. Globally significant antimicrobial-resistant clones identified by the Pneumococcal Molecular Epidemiology Network (PMEN) with different serotypes were selected [12]. Because we also wanted to assess the role of the capsule for survival of pneumococci in saliva, two unencapsulated strains (R6 and Spain^9V^-3 Δ*cps*) and two non-typeable strains (AHOY490 and AHOY2831) were included as well (Table 1).

**Table 1:** Bacterial strains, primers and plasmids used in this study.

Strain, primer or plasmid	Relevant features or nucleotide sequence (5′ to 3′)^a^	Reference/source or target gene^b^
***S. pneumoniae*** ** strains**		
Spain^6B^-2	Serotype 6B; PMEN No. 2	[13]
Spain^9V^-3	Serotype 9V; PMEN No. 3 (SP195)	[13]
Hungary^19A^-6	Serotype 19A; PMEN No. 6	[13]
South Africa^6B^-8	Serotype 6B; PMEN No. 8	[13]
Finland^6B^-12	Serotype 6B; PMEN No. 12	[13]
Poland^23F^-16	Serotype 23F; PMEN No. 16	[13]
Maryland^6B^-17	Serotype 6B; PMEN No. 17	[13]
Greece^6B^-22	Serotype 6B; PMEN No. 22	[13]
Colombia^23F^-26	Serotype 23F; PMEN No. 26	[13]
EF3030	Serotype 19F	[49]
TIGR4	Serotype 4	[50]
Δ*amiACDEF*	*amiACDEF* deletion mutant in strain Spain^9V^-3	This study
Δ*aroDEBC*	*aroDEBC* deletion mutant in strain Spain^9V^-3	This study
Δ*plsX*	*plsX* deletion mutant in strain Spain^9V^-3	This study
R6	Unencapsulated	[51]
Spain^9V^-3 Δ*cps*	Capsule (*cps*) deletion mutant of Spain^9V^-3	This study
AHOY490	Non-typeable	[52]
AHOY2831	Non-typeable	[52]
**Primers Deletion mutant construction**		
*amiACDEF*_L1	AGATCGTTTTAAGTCTGACGCAG	*amiACDEF*; left flank
*amiACDEF*_L2	CCACTAGTTCTAGAGCGGC CATGCTGCAAGTACACCTGC	*amiACDEF*; left flank
*amiACDEF*_R1	GAGAAGTCAAGAGGAGCCCC	*amiACDEF*; right flank
*amiACDEF*_R2	GCGTCAATTCGAGGGGTATC TTGTAGCAACGGTTCTGGCG	*amiACDEF*; right flank
*amiACDEF*_C	CAAGGTTTCTGGGTCTGCTG	*amiACDEF*; control
*aroDEBC*_L1	GGAGGAGCTAGCCAGACAAC	*aroDEBC*; left flank
*aroDEBC*_L2	CCACTAGTTCTAGAGCGGC AGCCTCCTCTAAACTTCTTGGC	*aroDEBC*; left flank
*aroDEBC*_R1	ACAAAGCGAACAGACTTGCC	*aroDEBC*; right flank
*aroDEBC*_R2	GCGTCAATTCGAGGGGTATC TTGTAGCAACGGTTCTGGCG	*aroDEBC*; right flank
*aroDEBC*_C	GAAAACCAACTCACGGCCTG	*aroDEBC*; control
*plsXSP195_0075*_L1	TGGAGTTGATGGAAGCAGGC	*plsXSP195_0075*; left flank
*plsXSP195_0075*_L2	CCACTAGTTCTAGAGCGGC ATGGCCTGAGGTGCGTAATC	*plsXSP195_0075*; left flank
*plsXSP195_0075*_R1	TCCAAATCTTGAATTGGCATCG	*plsXSP195_0075*; right flank
*plsXSP195_0075*_R2	GCGTCAATTCGAGGGGTATC CAGACTGCGCGTGAATTTTC	*plsXSP195_0075*; right flank
*plsXSP195_0075*_C	TGTCGCTGTCAGATATTGCTTG	*plsXSP195_0075*; control
**Mutant validation by qRT-PCR**		
*amiACDEF*_F	AGCATGGTTGACAGATAGTTCACTCT	*amiACDEF*; forward
*amiACDEF*_R	AATGGAACAATTCGTGAAAGCA	*amiACDEF*; reverse
*aroDEBC*_F	GGATGCCGACATCATTGAATG	*aroDEBC*; forward
*aroDEBC*_R	GGCTGGAGCTACCTGCAAAA	*aroDEBC*; reverse
*plsXSP195_0075*_F	GGAGATGAAGCTAAAATCAAGCAA	*plsXSP195_0075*; forward
*plsXSP195_0075*_R	TCATCATCCGAATCAATCTTCTCA	*plsXSP195_0075*; reverse
*gyrA*_F	AATGAACGGGAACCCTTGGT	*gyrA*; forward
*gyrA* _R	CCATCCCAACCGCGATAC	*gyrA*; reverse
**Plasmid**		
pGSF8	PCR2.1 with marinerT7-MmeI; Ap^r^, Km^r^, Sp^r^	[14]
**Tn-seq**		
PBGSF23	CAAGCAGAAGACGGCATACGAAGACCGGGGACTTATCATCCAACCTGT	GSF amplification primer 1
PBGSF29 ATCACG	TTCCCTACACGACGCTCTTCCGATCTATCACGNN	Adapter primer [14]
PBGSF30 ATCACG	P-CGTGATAGATCGGAAGAGCGTCGTGTAGGGAAAGAGT-P	Adapter primer [14]
PBGSF29 CGATGT	TTCCCTACACGACGCTCTTCCGATCTCGATGTNN	Adapter primer [14]
PBGSF30 CGATGT	P-ACATCGAGATCGGAAGAGCGTCGTGTAGGGAAAGAGT-P	Adapter primer [14]
PBGSF29 TTAGGC	TTCCCTACACGACGCTCTTCCGATCTTTAGGCNN	Adapter primer [14]
PBGSF30 TTAGGC	P-GCCTAAAGATCGGAAGAGCGTCGTGTAGGGAAAGAGT-P	Adapter primer [14]
PBGSF29 TGACCA	TTCCCTACACGACGCTCTTCCGATCTTGACCANN	Adapter primer [14]
PBGSF30 TGACCA	P-TGGTCAAGATCGGAAGAGCGTCGTGTAGGGAAAGAGT-P	Adapter primer [14]
PBGSF31	AATGATACGGCGACCACCGAGATCTACACTCTTTCCCTACACGACGCTCTTCCGATCT	GSF amplification primer 2 [14]

a Underlined sequences are complementary to primers used for amplification of antibiotic resistant cassettes.

b Left flank and right flank indicate positions relative to the target gene.

**Figure 2:**
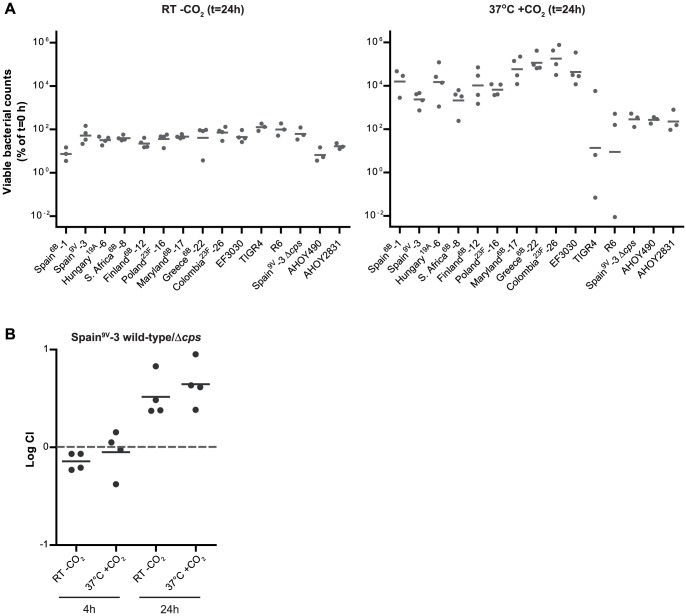
Single and competitive growth characteristics of *S. pneumoniae* strains in human saliva. A. Growth characteristics of several *S. pneumoniae* strains in human saliva. Strains were incubated in 100% saliva at RT without CO_2_ (left) or at 37°C with CO_2_ (right) for 24 h. Viable bacterial counts are presented as the percentage of the viable bacterial counts detected at t = 0 h. Each point represents a single measurement. Horizontal lines indicate geometric means. B. Competitive growth between wild-type Spain^9V^-3 and its unencapsulated derivative Spain^9V^-3 Δ*cps*. A 1∶1 ratio of the wild-type and its unencapsulated mutant was inoculated in 100% saliva at 37°C with CO_2_ and at RT without CO_2_. Competitive index scores (CI) were determined at t = 4 h and at t = 24 h. Experiments were performed in quadruplicate. Each point represents the log competitive index score of a single measurement. Horizontal lines represent the mean.

Undiluted human saliva was inoculated with 10^4^ CFU ml^−1^ *S. pneumoniae*. All tested *S. pneumoniae* strains survived in saliva at RT in CO_2_-poor ambient air and at 37°C in a CO_2_-rich environment (Figure 2A). At RT without CO_2_, most *S. pneumoniae* strains showed survival in saliva with geometric mean viable bacterial counts at t = 24 h around or below 100% of the bacterial starting inoculum (Figure 2A left). At 37°C with 5% CO_2_ all PMEN strains showed growth with geometric mean viable counts at t = 24 h at least 10-fold higher than the number of bacteria at t = 0 h. The TIGR4 strain showed survival rather than growth at 37°C with 5% CO_2_ with geometric mean CFU around 10% of the bacterial starting inoculum. The unencapsulated R6 strain also had a geometric mean viable count at t = 24 h around 10% of the bacterial starting inoculum. The unencapsulated derivative of Spain^9V^-3 (Spain^9V^-3 Δ*cps*) and the non-typeable AHOY490 and AHOY2831 strains showed growth at 37°C with CO_2_ with geometric mean viable counts around 250% of the starting inoculum (Figure 2A right).

We also evaluated the ability of the unencapsulated derivative of Spain^9V^-3 to survive and grow in saliva when in competition with its wild-type parent. While no difference was seen after 4 h, Spain^9V^-3 Δ*cps* outcompeted its encapsulated wild type after 24 h at both growth conditions (log CI>0, Figure 2B), demonstrating that the capsule is not essential for the survival in saliva.

Interestingly, all PMEN strains showed much better survival in saliva than the other strains tested at 37°C with 5% CO_2_, with the exception of EF3030. Since all PMEN strains tested were of the major clonal complex 156 and thus genetically related [13], their similarity in behavior in human saliva may have a genetic basis. The PMEN strains represent a group of important disease-causing pneumococci isolated from patients from different parts of the world [12]. It is tempting to speculate that the wide geographical distribution of the PMEN clones may be related to their increased ability to grow in human saliva and transmit through droplet spread. The epidemiological spread of PMEN clones is especially worrisome because these clones consist of penicillin-resistant and multidrug-resistant pneumococci and thus contribute to the increase in antimicrobial resistance worldwide [12].

### Identification of *S. pneumoniae* Spain^9V^ genes essential for growth and survival in saliva

To identify *S. pneumoniae* genes essential for growth and survival in saliva, we employed the genome-wide negative selection screen Tn-seq [11], [14]. To this end, a large random *S. pneumoniae* Spain^9V^-3 library was challenged by growth at RT in CO_2_-poor ambient air and at 37°C in 5% CO_2_. Examination of the relative abundance of each individual transposon mutant in the library before challenge (starting condition) by Tn-seq revealed transposon insertion mutants covered by at least 8 sequence reads in 26,289 unique TA-sites, representing a total of 1615 genes. Tn-seq data can be found on the ESSENTIALS website (http://bamics2.cmbi.ru.nl/websoftware/essentials/links.html).

**Figure 3:**
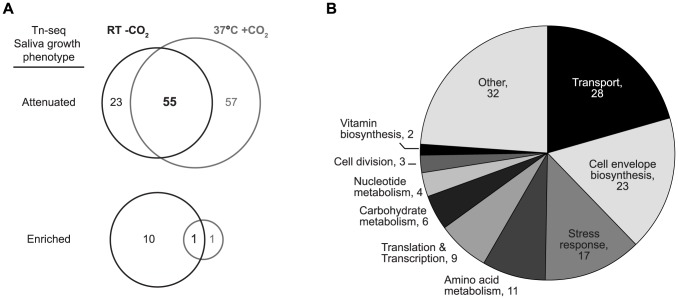
Genes affecting survival of *S. pneumoniae* in human saliva. A. Overlap between genes identified by Tn-seq as important for survival of *S. pneumoniae* in saliva at RT without CO_2_ or at 37°C with CO_2_. B. Biological properties and molecular functions of genes identified by the Tn-seq screen as essential for pneumococcal growth and/or survival in saliva. The numbers of genes within each category are indicated.

Our screen identified 78 genes potentially required for survival during 24 h in saliva at RT without CO_2_, as their corresponding mutants were negatively selected from the mutant-library during challenge (defined as at least 2-fold attenuated survival at 24 h compared with the starting library). After 4 h incubation at 37°C with 5% CO_2_, mutants of 112 genes were attenuated at least 2-fold. The overlap between genes involved in survival of *S. pneumoniae* in both conditions was substantial: 55 genes were identified as essential in both conditions (Figure 3A).

### Genes involved in cell envelope biosynthesis are essential for survival of *S. pneumoniae* in saliva

Functional annotation of the genes selected in either one or both of the challenge conditions (n = 135) revealed that, next to genes with an as yet unknown function, the two most important functional categories were transport and cell envelope biosynthesis (Table S1, Figure 3B). The pneumococcal cell envelope consists of a plasma membrane, the cell wall and the capsule. The cell wall is mainly composed of peptidoglycan (PG) and teichoic acids. Several genes identified as important for the survival of *S. pneumoniae* in saliva in our study contribute to resistance to antimicrobial peptides (AMPs) and lytic enzymes produced by the host, such as genes belonging to the *murMN* operon, *plsX* and *pgdA* (Table S1). In saliva, positively charged AMPs with membrane-damaging activity and antimicrobial PG lytic enzymes such as lysozyme constitute a first line of defense against infection [15]. Inactivation of *murMN* or the *pgdA* gene has been shown to result in hypersensitivity to lysis by lysozyme [16], [17]. We observed that mutants of *murMN* and *pgdA* were attenuated in saliva, probably because of their increased sensitivity to lysis by lysozome. Interestingly, pneumococcal cells in which *murMN* was inactivated completely lost penicillin resistance [18], [19]. This implies that inhibitors of *murMN* would not only potentially decrease survival of *S. pneumoniae* in saliva, they may also act as powerful synergists of penicillin against resistant *S. pneumoniae* strains.

Other attenuated mutants in the functional category of cell envelope biosynthesis included the ortholog of OatA, encoded by SP195_2011 in Spain^9V^-3 and by SP_2057 in TIGR4. OatA is responsible for O-acetylation of N-acetylmuramyl residues of peptidoglycan, forming N,6-O-diacetylmuramic acid. O-acetylation of the peptidoglycan is the major determinant for lysozyme resistance in *Staphylococcus aureus* [20] and a similar phenotype was observed for *S. pneumoniae* and *Lactococcus lactis oatA* mutants [21], [22]. Interestingly, the regulator Spx, encoded by SP195_A0021 in Spain^9V^-3 and by SP_0189 in TIGR4, was also found to be attenuated. Spx was found to be responsible for regulating the level of O-acetylation of peptidoglycan in *L. lactis* [22].

In pneumococci as well as in other gram-positive bacteria, the cell wall envelopes the plasma membrane which consists of phospholipids and various proteins. The *plsX* gene plays a role in the production of a fatty acid that is involved in membrane phospholipid synthesis in *S. pneumoniae* and represents a new target for the development of antibacterial therapeutics [23]. Since we observed that the *plsX* gene was important in survival of *S. pneumoniae* in human saliva, such new therapeutic agents may also play a role in the prevention of droplet transmission of *S. pneumoniae*. We hypothesize that the synthesis of fatty acids mediated by *plsX* is of extra importance for the maintenance of the membrane integrity in a fatty-acid poor environment, such as human saliva [24]. Interestingly, the ortholog of the regulator controlling fatty acid biosynthesis in *L. lactis* [25], encoded by SP195_0456 in Spain^9V^-3 and by SP_0416 in TIGR4, was also attenuated, suggesting that this regulator plays a similar role in *S. pneumoniae*.

Several other genes identified as essential for pneumococcal growth in saliva, such as *nagA* and *glmS*, are known key factors involved in cell wall biosynthesis in other bacteria [26], although their roles in *S. pneumoniae* are not yet fully understood.

### Genes involved in transport, stress response and amino acid metabolism are essential for survival of *S. pneumoniae* in saliva

The largest functional category for which genes were identified by the Tn-seq screening was transport while stress response and amino acid metabolism were respectively third- and fourth-largest (Figure 3B). In these three categories, as well as in the cell envelope biosynthesis category, several operons for which most genes were attenuated were identified, eg, the *amiACDEF* operon, the *mntABC* operon, the *pst* operon and the *aroDEBC* operon (Table S1). The *amiACDEF* operon encodes a multicomponent oligopeptide transporter in *S. pneumoniae* [27]. Oligopeptides are an important source of nutrients, but can also serve as signals for intercellular communication. In a murine model of pneumococcal colonization, deletion of *ami* genes resulted in pneumococci that were less able to colonize the nasopharynx [28]. While the oligopeptide transport systems are involved in the uptake of nutrients from the external environment, the *aro* genes are involved in the biosynthesis of aromatic amino acids [29]. Since the concentration of amino acids in saliva is low [15], their biosynthesis is likely to become more important, which explains the observed decrease in survival of the *aro* mutants in our screen.

The *pst* operon of *S. pneumoniae* encodes an ATP-binding cassette phosphate transporter [30]. The uptake of phosphate is of fundamental importance because phosphate is required as a nutrient in the cell physiology of *S. pneumoniae*. Mutants of *pst*, unable to transport phosphate over the cell membrane, are less able to take up available phosphate from the saliva which is a likely reason for their underrepresentation in human saliva.

Following these hypotheses concerning the potential roles of *plsX*, *amiACDEF*, *aroDEBC* and *pst* in pneumococcal survival and/or growth in saliva, we suggest that a limited presence of certain essential nutrients, such as fatty acids, oligopeptides and phosphate, in human saliva can result in a decreased resistance of *S. pneumoniae* to lytic enzymes when genes coding for nutrient biosynthesis or uptake are inactivated. The exact composition of human saliva varies between individuals and with changes in salivary flow [31]. Anti-pneumococcal antibodies may also be present in human saliva, especially in children [2], [3], and the influence of specific salivary components on the survival and transmission of *S. pneumoniae* through saliva warrants further investigation.

Manganese is relatively accessible in the human nasopharynx and has been measured at about 36 µM in saliva [32]. The *psa* operon encodes an ATP-binding cassette transporter for manganese uptake. In pneumococci, manganese transport by this receptor is important for pneumococcal competence and virulence [33], [34]. In addition, *psaA* mutant strains exhibit increased sensitivity to oxidative stress [35]. Considerable levels of antioxidants are found in human saliva, indicating the presence of oxidative stress in the oral cavity [36]. The uptake of manganese for protection against oxidative stress will thus be important for survival of *S. pneumoniae* in saliva, which may explain the decreased survival of mutants with a defect *mntABC* gene in our study. Although the concentration of manganese in saliva is considerable, it is restricted to nanomolar amounts at internal sites [32], [37], [38]. Manganese could thus be an important cue by which the immediate environment is sensed and the transitioning to internal body sites is experienced. Indeed, in clinical *S. pneumoniae* strains from Chinese patients, *psaA* expression was significantly higher in blood-derived than in sputum-derived *S. pneumoniae* [34]. Expression of *psaA* may thus be involved in invasion of the blood system by *S. pneumoniae*, making this gene a potentially interesting target for the development of new classes of antimicrobials.

Among the stress response genes, several genes that play a role in the chaperone system were identified, eg, *mecA*, *hslO*, *tigS* and *clpB* [39]–[42]. In eubacteria, the chaperone system and the chaperones form a functional network, which assists the folding of newly synthesized proteins [43]. Under stress conditions, unfolded and misfolded proteins may be generated, which escape this chaperone network and accumulate as protein aggregates. ClpB, a member of the HSP100/Clp family [44], has the capacity to facilitate the efficient disaggregation and refolding of a large variety of protein aggregates *in vitro* and *in vivo* [40], [45]. Possibly, saliva represents a stress condition under which misfolded proteins are generated. ClpB could thus be important for the refolding of protein aggregates, which explains its potential role in the survival of *S. pneumoniae* in saliva.

### Characterization of Tn-seq identified genes that are essential for survival of *S. pneumoniae* in saliva

For validation of the Tn-seq phenotypes, directed gene deletion mutants were generated in *S. pneumoniae* Spain^9V^-3 for representatives from the two main categories: *plsX* and *amiACDEF* (belonging to the categories of respectively cell envelope biosynthesis and transport) and for the largest operon for which multiple genes were selected in any of the other functional categories, ie, *aroDEBC*. Survival of these mutants in saliva under the two challenge conditions was assessed both during single growth, as well as in competition with the wild-type.

**Figure 4:**
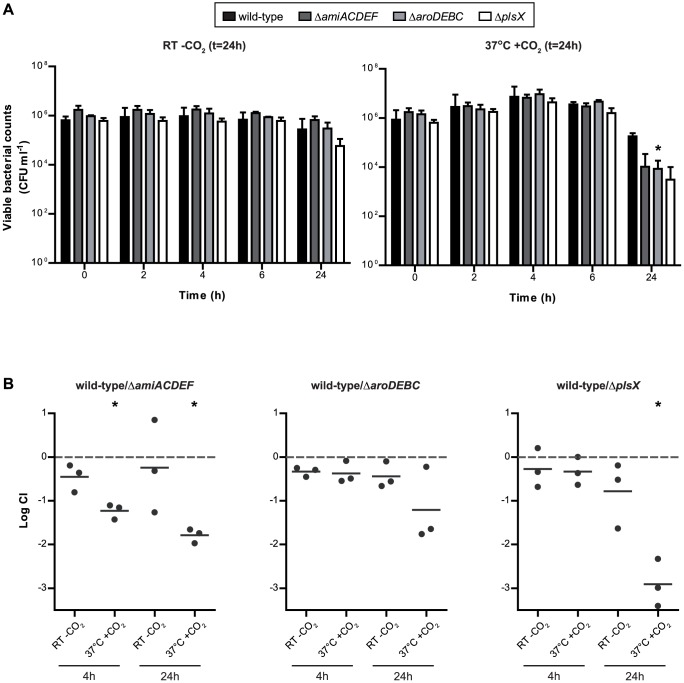
Survival of directed gene deletion mutants in human saliva. A. Single growth experiments. A starting concentration of 10^6^ CFU ml^−1^ wild-type or mutant bacteria was incubated with saliva at the two conditions. Median and interquartile range (IQR) of the CFU at each time point are displayed. After 24 h at 37°C with CO_2_, all mutant strains showed decreased survival, but this was only statistically significant for Δ*aroDEBC*. B. Competitive growth experiments. A 1∶1 ratio of the wild type strain and the mutant strain (5*10^5^ CFU ml^−1^ for each strain) was inoculated in saliva. Each black point represents the log competitive index score of an individual experiment. Values <0 indicate attenuation of the mutant strain. Horizontal lines represent the mean. After 24 h at 37°C with CO_2_, the wild-type strain outcompeted all mutant strains, but this was only statistically significant for Δ*amiACDEF* and Δ*plsX*.

In single culture experiments at RT without CO_2_, the *plsX* mutant showed a trend towards a decreased survival compared with the wild type at t = 24 (p = 0.056). The *amiACDEF* and *aroDEBC* mutant strains did not show significantly decreased survival compared to the wild type at any time point (Figure 4A). After 24 h at 37°C in a CO_2_-rich environment, all three mutant strains displayed decreased survival compared with the wild- type strain (Figure 4A). However, this was only significant for the *aroDEBC* mutant (p = 0.032).

Competition experiments showed that the wild-type strain outcompeted Δ*amiACDEF*, Δ*aroDEBC* and Δ*plsX* at t = 24 h at 37°C in a CO_2_-rich environment (Figure 4B) corroborating the Tn seq results, and this was statistically significant for Δ*amiACDEF* and Δ*plsX*. At RT without CO_2_, only the Δ*plsX* mutant outcompeted the wild-type at t = 24 h with a log CI score of −0.78, but this was not statistically significant. These results are in accordance with the Tn-seq screening, because mutants of the *plsX* gene showed attenuation at RT without CO_2_ with a log fold-ratio ≤−1.0, whereas mutants of *amiACDEF* and *aroDEBC* did not show attenuation at RT without CO_2_ (Table S1).

The finding that the competition assays confirmed the Tn-seq results, while no difference was observed between growth of the wild-type compared with the mutants in single culture experiments for *plsX* and *amiACDEF* can be explained by the fact that the Tn-seq screening is performed on a mutant library containing a diverse collection of single insertion mutants. The setup of the Tn-seq screening is therefore more comparable to the competition assay than to the single culture assay.

## Conclusions

In this study, we demonstrated that various clinically relevant *S. pneumoniae* strains showed both survival and growth in saliva under conditions representing in-host carriage (37°C with 5% CO_2_) and transmission (RT without CO_2_). Additionally, we applied the Tn-seq technology to gain insight into the genetic requirements of pneumococcal growth in saliva, and showed that particularly genes involved in cell envelope biosynthesis, transport, stress response and amino acid metabolism play a role in this process.

The observation that pneumococci survive and grow in human saliva underscores that *S. pneumoniae* can be transmitted via respiratory droplets. The results described in this study provide a basis for future investigations characterizing the survival and pathogenesis of *S. pneumoniae* in human saliva.

## Methods

### Ethics statement

Saliva from healthy adult volunteers was collected after obtaining verbal informed consent in the presence of the study team. Written informed consent was not obtained because sampling was non-invasive. The research ethics committee of the Radboud University Medical Centre approved the study protocol and informed consent procedure.

### Pneumococcal strains and growth conditions

All pneumococcal strains used in this study are shown in Table 1. *S. pneumoniae* Spain^9V^-3 (SP195, PMEN No. 3) was used for the generation of *S. pneumoniae* transposon mutant libraries since this was a PMEN strain and thus isolated from patients with pneumococcal disease from several parts of the world [12]. Furthermore, the Spain^9V^-3 strain showed survival after 24 h at RT and growth after 24 h at 37°C with geometric mean viable bacterial counts at t = 24 h around or above 100% of the bacterial starting inoculum (Figure 2). *S. pneumoniae* strains were routinely grown in Brain Heart Infusion (BHI) broth unless indicated otherwise or on blood agar (BA) plates composed of Columbia agar (Oxoid, Hampshire, United Kingdom) supplemented with 5% defibrinated sheep blood (Biotrading, Mijdrecht, Netherlands). All cultures were incubated in a 5% CO_2_ incubator at 37°C. *S. pneumoniae* freezer stocks were prepared from cultures grown to mid-log phase until an optical density at 620 nm (OD_620_) between 0.2 and 0.3 and stored with 15% glycerol at -80°C. Viable bacterial counts were determined by plating serial 10-fold dilutions in PBS on BA plates. *S. pneumoniae* mutant libraries and directed mutants were selected with BA plates containing 150 µg ml^−1^ spectinomycin.

### Generation of *S. pneumoniae* transposon mutant libraries

*S. pneumoniae* Spain^9V^-3 *mariner* transposon mutant libraries were generated essentially as described previously [46]. Briefly, 1 µg of pneumococcal genomic DNA was incubated in the presence of purified *Himar1* transposase and 1 µg of plasmid pGSF8 as a donor of the *mariner* transposon conferring spectinomycin resistance. After repair of the resulting transposition products, 100 ng mutagenized DNA was used for transformation of 100 µl competent *S. pneumoniae* cells. For mutant libraries, the required number of colonies was scraped from the plates, pooled, grown to mid-log phase and stored in 15% glycerol at −80°C.

### Identification of genes essential for *S. pneumoniae* survival in and transmission through saliva by Tn-seq analysis

For the Tn-seq screen, the *S. pneumoniae* Spain^9V^ transposon mutant library was first pre-cultured until mid-log phase. Approximately 10^6^ CFU of the mutant library were inoculated into 1 ml saliva, and incubated either at 37°C with 5% CO_2_ for 4 h or at RT without CO_2_ for 24 h (n = 4 for each condition). After challenge, samples were supplemented with 15% of glycerol and stored at −80°C. Equivalent CFUs of the recovered libraries and the start library were expanded to mid-log, and chromosomal DNA was isolated using Genomic-tip 20/G columns.

### Tn-seq data analysis

The Tn-seq technology was used to profile the relative abundance of each mutant in the library before and after challenge as described previously [14]. For Tn-seq data analysis, FASTQ files were processed via the ESSENTIALS data analysis pipeline (http://bamics2.cmbi.ru.nl/websoftware/essentials/essentials_start.php) [47]. Tn-seq bar code sequences were used to attribute sequence reads to individual samples. Sequence reads were aligned to the Spain^9V^-3 genome (accession number NZ_ABGE00000000) with a minimum match of 16 nucleotides and collected per unique insertion site (TA dinucleotide sequence) and per gene. The number of unique transposon insertion sites was defined as the positions that had at least 8 “pseudo” sequence reads in the initial “start” library after normalization. In total 26,289 unique mutants where found. Selection criteria for genes of which mutants were affected by the presence of saliva were as follows: (1) a log_2_ fold-change in sequence reads between the start library and the library challenged with saliva (either for 24 h at RT or for 4 h at 37°C+5%CO_2_) ≤−1 or ≥1 and a Benjamini-Hochberg adjusted p-value <0.05; (2) a minimum number of reads in the start library of 100; and (3) a fitness score >−3.5, with the fitness score defined as the ratio between actual measured sequence reads and expected sequence reads based on possible TA-insertions sites and library size [47].

Genes were assigned to the functional categories transport, cell envelope biosynthesis, stress response, amino acid metabolism, translation and transcription, carbohydrate metabolism, nucleotide metabolism, cell division and vitamin biosynthesis based on the involvement of their encoded proteins or their orthologs in these processes.

### Construction of *S. pneumoniae* deletion mutants

Directed-deletion mutants of *S. pneumoniae* were generated by allelic exchange of the target gene with an antibiotic resistance marker as described previously using a megaprimer polymerase chain reaction (PCR) method [46]. In short, an extension PCR was performed to join 400–500 bp 5′and 3′ flanking sequences of the target gene with the spectinomycin resistance cassette (obtained from pR412T7), which was subsequently transformed into naturally competent *S. pneumoniae* Spain^9V^-3 bacteria grown in CTM medium. Next, 100 ng of DNA of the first-generation directed mutants was crossed back into the *S. pneumoniae* Spain^9V^-3 wild-type strain and selected on spectinomycin plates. At the same time, competent cells were also processed through the transformation procedure without the addition of DNA to obtain a coupled wild-type strain. All primers used in this study (obtained from Biolegio, Nijmegen, The Netherlands) are shown in Table 1.

Deletion of genes from mutants was confirmed by quantitative real-time polymerase chain reaction (qRT-PCR) expression analyses. To this end, strains were grown to OD_620nm_ ∼0.5 in THY medium and RNA was isolated as previously described [48]. DNA-free total RNA (500 ng) was reverse transcribed into cDNA using the iScript kit (Bio-Rad). qRT-PCR was performed using SYBR green chemistry on a CFX-96 real-time PCR machine (Bio-Rad). For all mutants, expression of the deleted gene(s) was abolished, while expression levels of the reference gene *gyrA* were comparable to the wild-type.

### Saliva collection

Saliva from healthy volunteers was collected in the early morning after overnight fasting from 11 pm the night before. Oral hygiene was performed not closer than three hours before collection of saliva. After collection, the saliva of at least two donors was pooled, put on ice and centrifuged at 16,000 g and 4°C for 15 minutes. The supernatant was sterilized by ultrafiltration with 0.45 µm filters (Whatman, Dassel, Germany). The sterility of the saliva was verified by incubation of a small portion on BA plates for 48 h at 37°C.

### Evaluation of the survival and competitive growth of *S. pneumoniae* strains in human saliva

For survival assay analyses, a starting concentration of 10^6^ CFU ml^−1^ bacteria was incubated with saliva at two conditions: 37°C with 5% CO_2_ and room temperature (RT) without CO_2_. At t = 0, t = 2, t = 4, t = 6 and t = 24 h samples were taken for CFU count. For competition experiments, a 1∶1 ratio of the wild type strain and the mutant strain (5*10^5^ CFU ml^−1^ for each strain) was inoculated in saliva. At the same time points as those used for the single cultures, the total number of bacteria was enumerated by plating serial dilutions on blood agar plates and the number of mutant bacteria was enumerated by plating serial dilutions on spectinomycin plates. Experiments were performed in triplo, quadruple or pentuple.

For competition experiments, a competitive index (CI) score was calculated as the output ratio of the viable bacterial counts of mutant to wild type bacteria divided by the input ratio of mutant to wild type bacteria. A log CI score of <0 indicates that the mutant is outcompeted by the wild type. For samples in which no viable bacteria were recovered, the lower limit of detection (222 CFU ml^−1^) was substituted as the numerator.

### Statistical analysis

Statistical differences in growth of *S. pneumoniae* strains were assessed using unpaired Student's t tests or the nonparametric Mann-Whitney's tests depending on whether or not the log10 transformed values of viable bacterial counts were normally distributed (Kolmogorov-Smirnov's test, p>0.05). Statistical differences in growth of *S. pneumoniae* in multiple dilutions of saliva were assessed by a one-way analysis of variance (ANOVA) and Tukey post hoc tests. For the competitive growth experiments, a one-sample *t* test on log-transformed CI scores (with an arbitrary mean of 0 and a p-value of <0.05) was used to calculate statistical significance.

## Supporting Information

Table S1 **Genes of** ***S. pneumoniae*** **of which transposon mutants showed attenuated or enhanced survival and/or growth in human saliva, categorized by functional class.**
(XLSX)
